# Functional Evolution of Leptin of *Ochotona curzoniae* in Adaptive Thermogenesis Driven by Cold Environmental Stress

**DOI:** 10.1371/journal.pone.0019833

**Published:** 2011-06-16

**Authors:** Jie Yang, Timothy G. Bromage, Qian Zhao, Bao Hong Xu, Wei Li Gao, Hui Fang Tian, Hui Jun Tang, Dian Wu Liu, Xin Quan Zhao

**Affiliations:** 1 Department of Epidemiology and Statistics, School of Public Health, Hebei Medical University, Shijiazhuang, Hebei, People's Republic of China; 2 Department of Biomaterials and Biomimetics, Department of Basic Science and Craniofacial Biology, New York University College of Dentistry, New York, New York, United States of America; 3 Graduate School of the Chinese Academy of Sciences, The Chinese Academy of Sciences, Beijing, People's Republic of China; 4 Microbiology Department, Shijiazhuang Center for Disease Control and Prevention, Shijiazhuang, Hebei, People's Republic of China; 5 Key Laboratory of Qinghai-Tibetan Plateau Biological Evolution and Adaptation, Northwest Plateau Institute of Biology, The Chinese Academy of Sciences, Xining, Qinghai, People's Republic of China; University of South Florida, United States of America

## Abstract

**Background:**

Environmental stress can accelerate the directional selection and evolutionary rate of specific stress-response proteins to bring about new or altered functions, enhancing an organism's fitness to challenging environments. Plateau pika (*Ochotona curzoniae*), an endemic and keystone species on Qinghai-Tibetan Plateau, is a high hypoxia and low temperature tolerant mammal with high resting metabolic rate and non-shivering thermogenesis to cope in this harsh plateau environment. Leptin is a key hormone related to how these animals regulate energy homeostasis. Previous molecular evolutionary analysis helped to generate the hypothesis that adaptive evolution of plateau pika leptin may be driven by cold stress.

**Methodology/Principal Findings:**

To test the hypothesis, recombinant pika leptin was first purified. The thermogenic characteristics of C57BL/6J mice injected with pika leptin under warm (23±1°C) and cold (5±1°C) acclimation is investigated. Expression levels of genes regulating adaptive thermogenesis in brown adipose tissue and the hypothalamus are compared between pika leptin and human leptin treatment, suggesting that pika leptin has adaptively and functionally evolved. Our results show that pika leptin regulates energy homeostasis via reduced food intake and increased energy expenditure under both warm and cold conditions. Compared with human leptin, pika leptin demonstrates a superior induced capacity for adaptive thermogenesis, which is reflected in a more enhanced β-oxidation, mitochondrial biogenesis and heat production. Moreover, leptin treatment combined with cold stimulation has a significant synergistic effect on adaptive thermogenesis, more so than is observed with a single cold exposure or single leptin treatment.

**Conclusions/Significance:**

These findings support the hypothesis that cold stress has driven the functional evolution of plateau pika leptin as an ecological adaptation to the Qinghai-Tibetan Plateau.

## Introduction

The Qinghai-Tibetan Plateau is the largest and highest plateau in the world with an average altitude more than 4000 m. Uplift of the Qinghai-Tibetan Plateau has had a great impact on the environment, resulting in selection on its organisms for a variety of functional adaptations to this unique ecological environment. Cold and hypoxia are the two most remarkable climatic properties on the Qinghai-Tibetan Plateau. Over their evolutionary histories, indigenous animals have formed their own adaptive strategies in response to these climatic extremes on the Plateau [1]. Plateau pikas (*Ochotona curzoniae*), also called the “black-lipped pika”, are small, non-hibernating and diurnal lagomorphs inhabiting the alpine meadows of the Qinghai-Tibetan Plateau. They are the keystone species in the Plateau ecosystem and are a “gold standard” mammal in the research areas of cold and hypoxia adaptation [1], [2]. Compared with other small mammals residing in tropical and subtropical regions, pikas have an unusually high resting metabolic rate (RMR) and non-shivering thermogenesis (NST) as an adaptation to the extreme cold on the Qinghai-Tibetan Plateau [3].

Leptin is an adipocyte-secreted hormone with an important role in the regulation of adaptive thermogenesis to cope with environmental temperature changes [4]. Leptin administration can increase energy expenditure mainly via the activation of sympathetic nerve activity and the turnover of norepinephrine in brown adipose tissue (BAT) [5], [6]. NST by BAT is one of the most important strategies that small mammals may use to defend hypothermia during a cold challenge. Adaptive recruitment of NST capacity by BAT may contribute to more than 60–70% of total NST during chronic cold exposure. Leptin has been considered as one of the regulators of NST in BAT [7], [8].

Because of the extreme Plateau climate and pika energy metabolism under such cold conditions, it is reasonable to hypothesize that leptin, acting as a cold stress-response protein, plays an important role in pika adaptation to the harsh environment. In our previous investigations, the leptin of plateau pika was cloned and observed to exhibit divergent mRNA expression levels with different altitudes and seasons [9], [10]. Molecular evolution analysis further suggested that positive selection may have acted on pika leptin [11]. Accordingly, we put forward the hypothesis that adaptive evolution has occurred in pika leptin in response to extreme cold.

To test the hypothesis that pika leptin is the result of adaptive evolution to extreme cold, it is necessary for us to investigate its physiological relationship to BAT-mediated thermogenesis. Therefore, in the present study we first express recombinant active pika leptin purified from an *Escherichia coli* system. Then, characteristics of adaptive thermogenesis of pika leptin under both warm and cold acclimation are determined. Finally, expression levels of genes related to thermogenesis are compared between pika leptin-treated and human leptin-treated mice to identify whether or not our hypothesis concerning the functional evolution of pika leptin in regulating thermogenesis is supported.

## Results

### Expression and purification of recombinant Plateau pika leptin and human leptin

**Figure 1:**
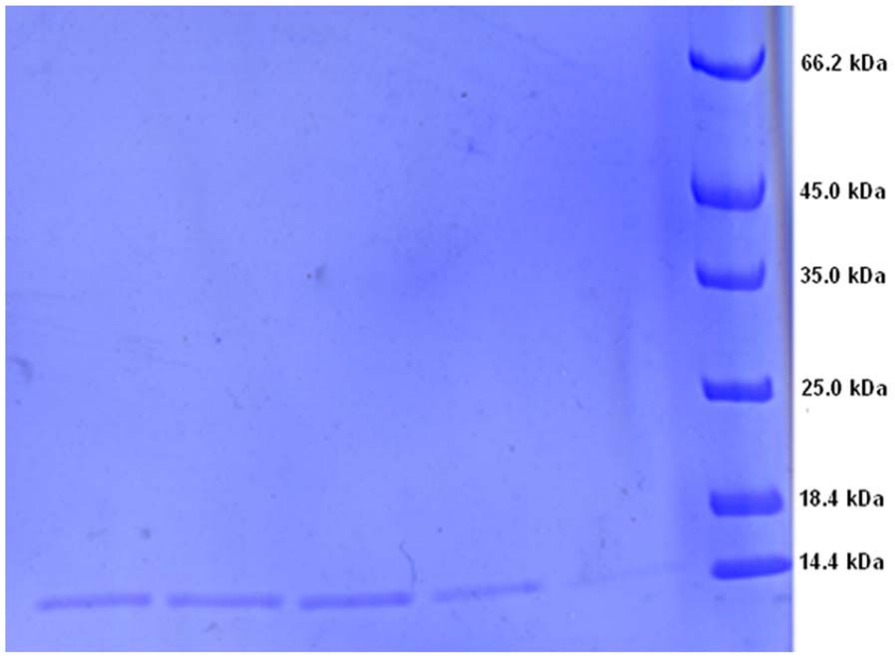
Purified recombinant plateau pika leptin.

Leptin from different species are frequently expressed and successfully purified using *Escherichia coli* as host because of its ease of use, rapid cell growth, low cost of culturing and well documented protocols. However, due to over-expression of heterologous protein in *E.coli*, most recombinant leptin proteins are, in general, expressed into an inactive form, and soluble recombinant leptin levels are inadequate. Thus, using this model system, recombinant leptin proteins need to be refolded to restore biological activity. To seek a strategy for obtaining adequate amounts of expressed soluble of recombinant plateau pika leptin, we optimized expression conditions including culture temperature, induction concentration of IPTG (Isopropyl-β- -thiogalactopyranoside), *E.coli* strains, and expression vectors with different fusion tags. cDNA of mature protein without signal peptide (441 bp) was cloned into pMAL-c2x vector and expressed as a partly soluble fraction accounting for 60 percent of total protein in lysate of *E.coli*. Recombinant pika leptin protein was purified directly via Immobilized Metal Affinity Chromatography (IMAC). The resulting recombinant pika leptin exceeded 90 percent of total protein composition. Figure 1 shows the purified recombinant plateau pika leptin. We obtained recombinant human leptin protein via the same production process as that used for obtaining pika leptin.

### Reduction in food intake and body weight after pika leptin administration under both warm and cold acclimation

**Figure 2:**
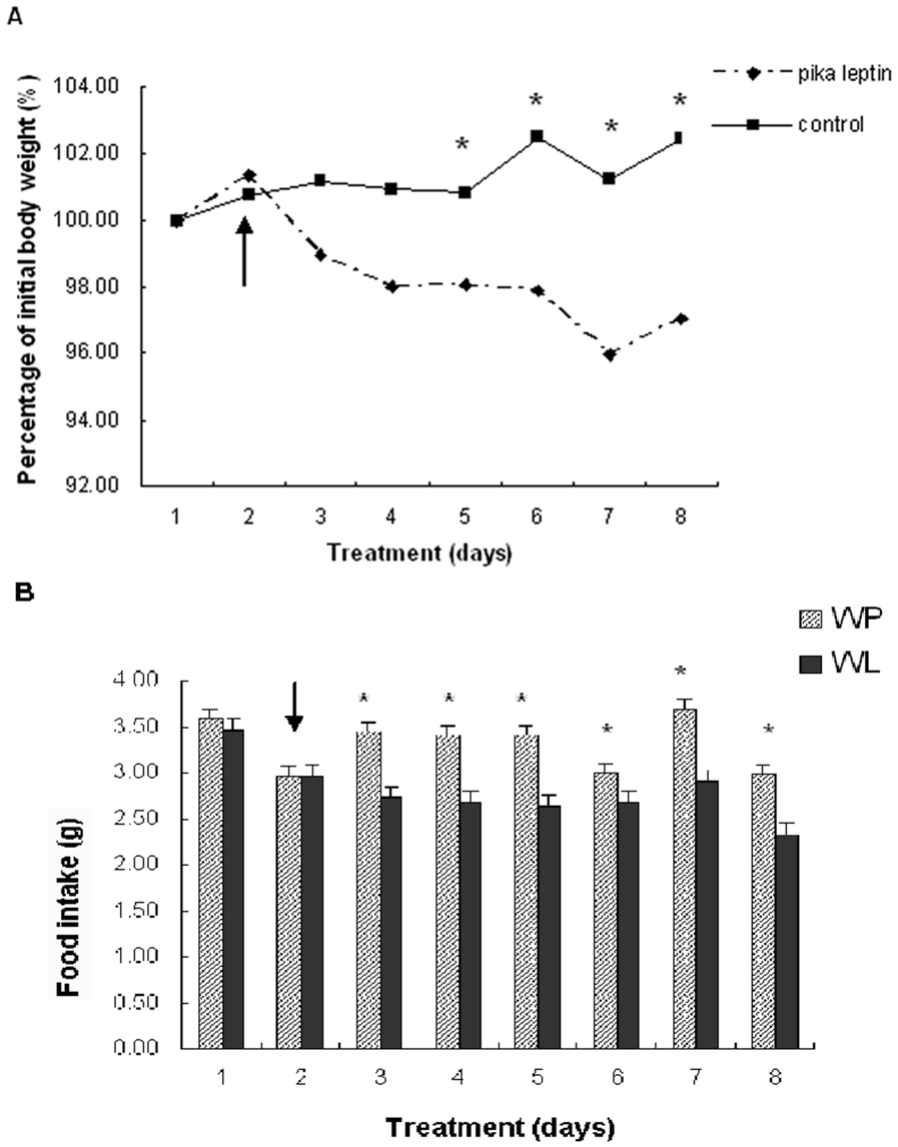
Effects of pika leptin administration on body weight (A) and food intake (B) under warm acclimation. Body weight is expressed as the percentage of body weight recorded one day prior to the onset of treatment. WP group, given PBS infusion; WL group, given leptin infusion. (n = 8 per group). Arrowhead points to the onset of infusion. The symbol * indicates significant differences (p<0.05) between WP group and WL group in body weight and food intake.

Under warm acclimation conditions (23±1°C), the pika leptin-treated mice lost 0.86 g of body weight during 7 days of treatment, whereas the PBS-treated mice on *ad libitum* feeding gained 0.31 g. A decrease in the body weight pika leptin-treated mice was observed, becoming significantly different (p<0.05) from PBS-treated mice by the 4^th^ day of injection (Figure 2A and Table 1). Food intake was also reduced to 2.33 g/day in pika leptin-treated mice compared to 2.97 g/day in PBS-treated mice (p<0.05; Figure 2B and Table 1). Prolonged cold exposure (5±1°C) had obvious effects on energy homeostasis as observed by increased food intake by CP mice (cold exposed mice receiving PBS buffer) compared to WP mice (warm exposed mice receiving PBS buffer) (WP: 3.60 g/day vs. CP: 4.20 g/day, p<0.05; Table 1). Under cold exposure pika leptin also significantly reduced food intake in CL mice (cold exposed mice receiving leptin treatment) compared to CP mice (cold exposed mice receiving PBS buffer) (CL: 3.48 g/day vs. CP: 4.12 g/day, p<0.05) and consequently body weight as well (reduction of body weight over 7 days, CL: 0.33 g vs. CP: 1.84 g; Table 1). Body length in leptin-treated mice measured 18.5±2.3 cm vs. 18.2±3.3 cm in PBS-controls (not significant, NS).

**Table 1:** Metabolic profiles of C57BL/6J mice treated with PBS and recombinant pika leptin under different temperatures.

Parameters	23±1°C (7 days)^a^				5±1°C (7 days)^b^			
	PBS		Pika leptin		PBS		pika leptin	
	day 0	day 7	day 0	day 7	day 0	day 7	day 0	day 7
Body weight (g)	20.45±1.82	20.76±1.58	19.76±1.29	18.90±1.20*	20.22±1.89	19.89±1.78	20.52±1.30	18.68±1.99^++^
Food intake (g/day)	3.60±0.12	2.97±0.33	3.48±1.41	2.33±0.40^a^	4.20±0.24	4.12±0.57	4.35±0.78	3.48±0.35^++^
Organ weight								
Heart / body weight (mg/g)		4.81±0.69		4.79±0.64		7.00±0.60		7.14±0.68
Lung / body weight (mg/g)		7.30±0.99		7.66±0.77		6.56±0.66		6.96±0.75
Liver / body weight (mg/g)		43.60±4.62		43.98±5.01		52.40±4.86		52.10±7.75
Spleen / body weight (mg/g)		7.18±1.26		7.33±1.47		3.70±0.84		6.23±0.68^++^
Kidney / body weight (mg/g)		7.06±0.88		6.37±0.97		9.44±1.25		9.62±1.17
White adipose tissue / body weight (mg/g)		4.27±1.21		3.54±1.31		3.32±1.58		0.61±0.16^++^
Brown adipose tissue / body weight (mg/g)		3.00±0.39		1.95±0.39*		3.67±0.46		2.53±0.07^++^
Parameters in plasma								
Blood glucose (mg/dL)		148.97±46.33		88.68±30.38*		116.39±23.20		74.26±25.24^++^
Plasma Triglyceride (mg/dL)		57.32±7.75		44.72±8.82*		46.80±19.28		31.85±19.95
Plasma Cholesterol (mg/dL)		242.82±69.18		164.16±26.23*		94.23±28.09		46.75±16.34^++^
Plasma Free fatty acids (mM)		0.46±0.12		0.28±0.10*		0.70±0.17		0.47±0.09^++^
Parameters in BAT								
Triglyceride content (mg/g tissue)		56.25±12.85		13.44±2.50*		92.70±25.27		52.78±19.78^++^
Mitochondrial protein content (µg/mg tissue)		1.52±0.79		2.34±0.75*		2.11±1.00		3.93±1.00^++^

a After acclimation at 23±1°C for 7 days, the mice were treated at 23±1°C for 7 days,named as WL (pika leptin-treated) and WP (PBS-treated), respectively.

b After cold acclimation (5±1°C) for 28 days, the mice were treated for 7 days at 5±1°C, named as CL(pika leptin-treated) and CP(PBS-treated), respectively.

*Significant difference between PBS and pika leptin groups at day 7 at 22±2°C.

++Significant difference between PBS and pika leptin groups at day 7 of treatment at 5±1°C after chronic cold acclimation for 28 days.

### Effects of pika leptin administration on body compositions of mice under warm and cold acclimation

**Figure 3:**
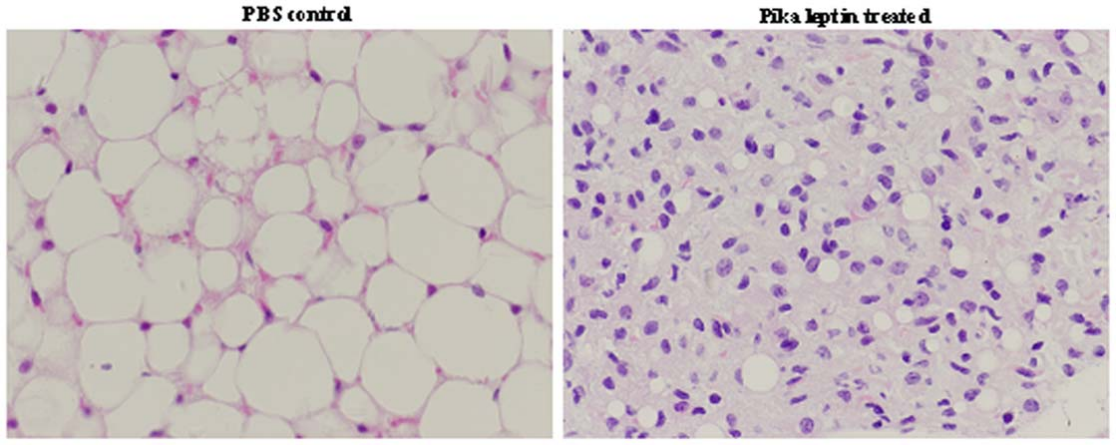
Histomorphology of white adipose tissue from pika leptin-treated and PBS-treated mice. Hematoxylin/eosin staining of WAT sections from PBS control group and pika leptin-treated group.

To further explore the reason pika leptin induced weight loss, we weighed the organs of mice exposed to different temperature conditions. Both pika leptin treatment and chronic cold exposure had significant effects on body composition, which presented as a distinct loss in body fat mass and not in lean body mass. White adipose tissue (WAT) weight ((WAT weight / total body weight) ×1000) decreased in warm and cold exposed mice receiving pika leptin (WL and CL respectively) compared to warm and cold exposed mice receiving PBS control (WP and CP respectively) (3.54±1.31 vs. 4.27±1.21 mg/g, WL vs. WP; 0.61±0.66 vs. 3.32±1.58 mg/g, CL vs. CP, p<0.05). Histomorphology of WAT confirmed that white adipocytes of mice treated by pika leptin exhibited a marked decrease in intercellular lipid accumulation; smaller and fewer lipid droplets than those from PBS control mice (Figure 3). BAT was another target organ affected by both pika leptin and cold exposure. Pika leptin treatment distinctly reduced BAT weight ((BAT weight/total body weight) ×1000) compared to PBS-controls under both warm and cold temperatures (1.95±0.39 vs. 3.00±0.39, WL vs. WP, p<0.05; 2.53±0.07 vs. 3.67±0.46, CL vs. CP, p<0.05). However, chronic cold exposure increased BAT weight (3.00±0.39 vs. 3.67±0.46, WP vs. CP, p<0.05, table 1). Interestingly, cold exposure also contributed to a reduction in spleen weight (WP: 7.18±1.26 mg/g vs. CP: 3.70±0.84 mg/g; p<0.05; Table 1), whereas pika leptin could restore normality of spleen weight (CP: 3.70±0.84 mg/g vs. CL: 6.23±0.68 mg/g; p<0.05; Table 1). However, under warm acclimation spleen weight did not obviously change (Table 1). The mass of heart, lung and kidneys was not affected by leptin treatment or cold exposure (table 1). These findings suggest that the primary target tissues of pika leptin are on body fat and not lean body mass.

### Decreased lipid accumulation and increased mitochondrial biogenesis in BAT after pika leptin treatment

**Figure 4:**
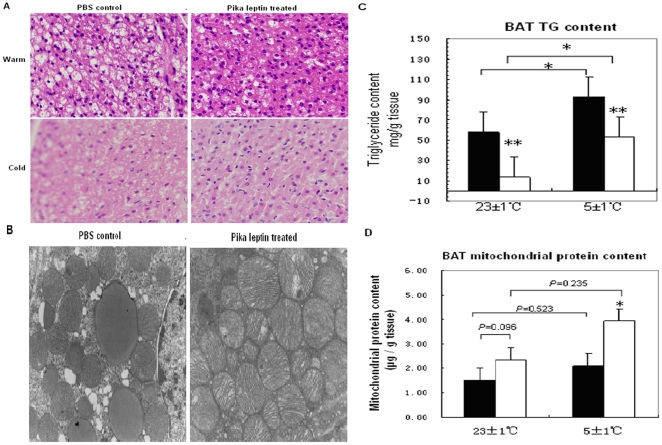
Characteristics of BAT from different treatments under both warm and cold acclimation. (A) Hematoxylin / eosin staining of BAT sections from PBS-control and pika leptin-treated mice under both warm and cold acclimation. (B) Representative electron microscopy images of BAT of PBS-control and pika leptin-treated mice under cold acclimation. (C) Triglyceride content in BAT of PBS-control and pika leptin-treated mice under both temperatures. (D) Mitochondrial protein mass in BAT of PBS-control and pika leptin-treated mice under both temperatures. Black bar indicates the PBS control group and the white bar indicates the pika leptin group.

Due to the crucial role of BAT in mammals' adaptive thermogenesis, we next explored the effects of pika leptin on thermogenesis in BAT under different temperatures. Histomorphological analysis of BAT confirmed that brown adipocytes treated with pika leptin exhibited a remarkable decrease in intercellular lipid accumulation, characterized by smaller and fewer lipid droplets compared to PBS control mice. Under cold acclimation, there was also a high density of capillary vessels in the two groups, especially obvious in the pika leptin treated group (Figure 4A). The ultrastructure of brown adipocytes was further observed via transmission electron microscopy. The images revealed that brown adipocytes treated with pika leptin exhibited a significant increase in intracellular density of mitochondria and a marked decrease in intracellular accumulation of triglycerides (TG) (Figure 4B). Measurement of the triglyceride content in BAT showed a significant reduction with pika leptin treatment compared to the PBS control under both temperatures. Cold exposure could induce an increase in TG content of BAT in both groups (56.25±12.85 mg/g tissue vs. 92.70±25.27 mg/g tissue, WP vs. CP, p<0.05; Figure 4C and Table 1). Furthermore, mitochondria were purified from whole BAT and protein content was confirmed. We found that pika leptin treatment caused an increase in mitochondrial protein content, which was more obvious under cold than warm conditions (increased percent of protein content compared to PBS control: 53.9% vs. 86.3%, warm vs. cold acclimation condition, p<0.05, Figure 4D and Table 1). The above findings suggest that pika leptin decreases lipid accumulation and increases mitochondrial biogenesis in BAT, especially significant when exposed to cold.

### Expression differences of genes related to adaptive thermogenesis between pika leptin and human leptin under warm and cold acclimation

**Figure 5:**
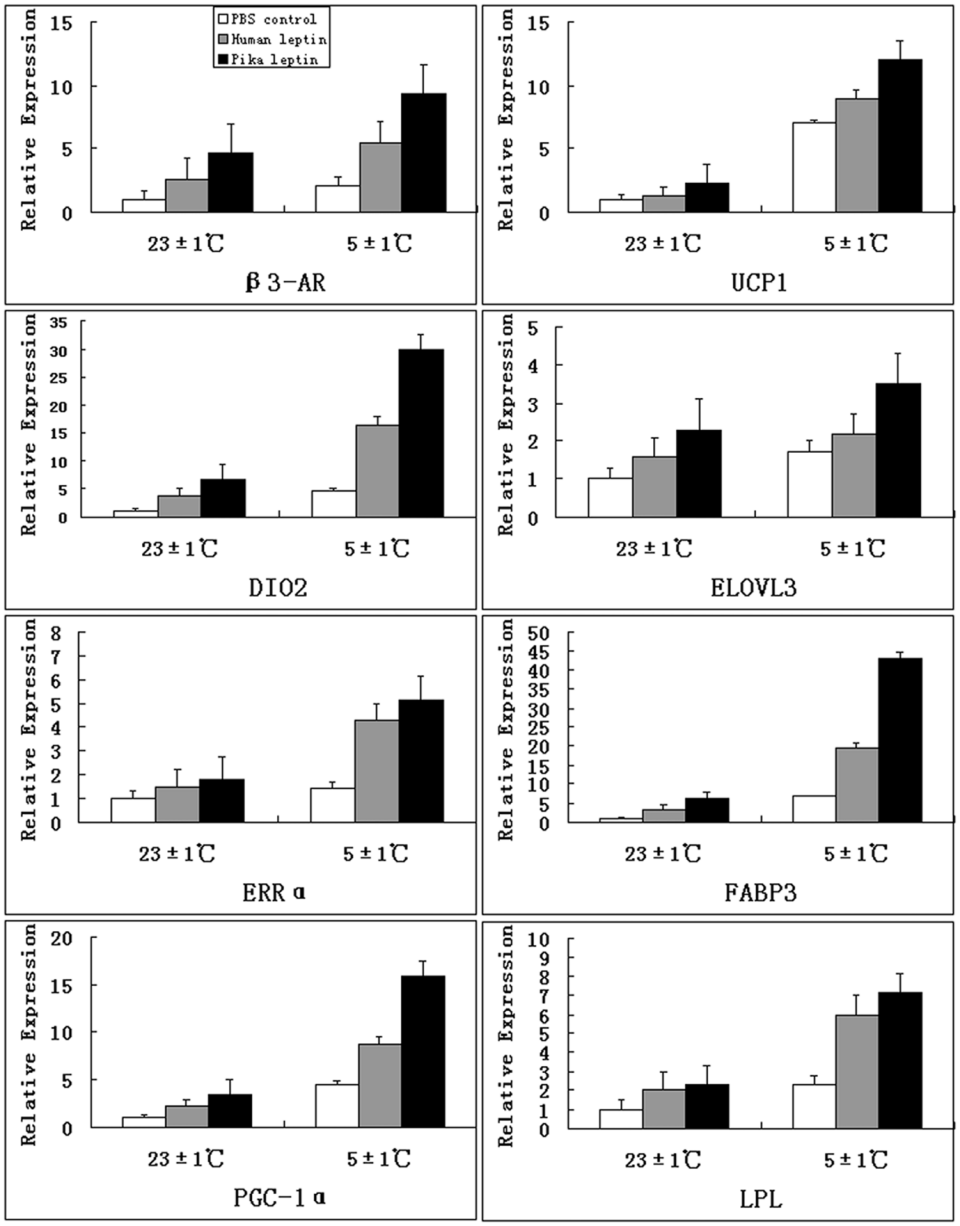
Comparison of expression level of hallmark genes involved in adaptive thermogenesis in brown adipose tissue.

**Figure 6:**
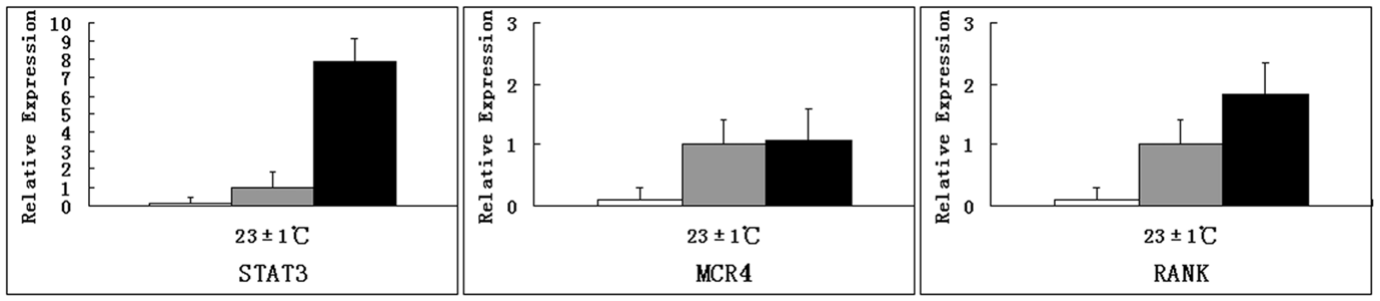
Comparison of expression level of key genes involved in adaptive thermogenesis in hypothalamus.

To evaluate the characteristics and mechanisms of adaptive thermogenesis of pika leptin in vivo, mRNA expression levels of genes with roles in mitochondrial energy metabolism in BAT and the hypothalamus were compared between pika leptin and human leptin groups under both warm and cold acclimation. Expression levels of the BAT-specific thermogenic genes,such as UCP1, ERRα, PGC-1α, FABP3, β3-AR, DIO2 and ELOVL3 were increased by 19%∼97% in pika leptin compared to human leptin under both warm and cold temperatures (Figure 5). Under warm condition, an obvious difference in the expression levels of thermogenic genes in hypothalamus such as STAT3, MCR4 and RANK were observed between pika leptin and human leptin (Figure 6). There was particularly increased expression in STAT3 with about 7.9 fold elevation and slight up-regulation of MCR4 expression in pika leptin compared to human leptin. The expression level of RANK was increased by 83% in pika leptin. Compared with warm conditions, cold exposure also affected expression levels of these genes and led to ≈1.4 to 7 fold increased expression level in BAT in PBS-treated mice. In sum, both cold exposure and pika leptin treatment affect expression levels of genes involved in thermogenesis. Furthermore, physiologic roles of pika leptin in adaptive thermogenesis were superior to human leptin, suggesting that pika leptin has adaptively and functionally evolved.

## Discussion

Adaptation to environmental variability and stress is essential for organism survival. Thermoregulation is particularly important for animals under cold conditions. The harsh cold and hypoxic climate of the Qinghai-Tibetan Plateau is a natural selection force operating to select for a specific adaptative strategy for plateau pikas, including a high thermogenetic capacity and metabolism to cope with these stresses. The adaptive evolution of such a phenotype has taken advantage of the variation of genes that increase fitness to this stressful environment [12], [13]. In a previous investigation, the hypothesis that natural selection may act on pika leptin was formulated on the basis of sequence analysis and inferences drawn from molecular evolution [11]. However, to date experimental demonstration of adaptive functional evolution in pika leptin have not performed. In present study, therefore, recombinant pika leptin was produced and its functions with regard to thermogenesis were examined under both warm and cold conditions.

Leptin, acting as a “satiation signal” to sense energy demand for different physiological states, is well documented to suppress food intake, reduce body weight and to increase energy expenditure in most studies [4]. In the present study, pika leptin confirmed these findings, obviously decreasing food intake and body weight from the second day of pika leptin injection till the end of experiment compared to PBS control. The target tissue that pika leptin acts upon are identified as fat body mass including white adipose tissue and brown adipose tissue, and not lean mass. Light microscope observations reveal changes in adipose tissue to include significantly smaller size and reduced droplet accumulation in both white and brown adipocytes. Temperature also directly affects fat body mass. Compared with warm condition, there is a clearly reduced lipid droplet accumulation but enhanced capillary vessel density in BAT under cold acclimation, especially enhanced in the pika leptin treated group. Dense vascularization ensures a sufficient supply of oxygen and metabolic substrates to be utilized by BAT, which indicates enhanced activation of BAT function. White and brown adipose tissues are key organs for the regulation of energy homeostasis, which have completely opposite functions. WAT accumulates surplus energy mainly in the form of triacylglycerol (TG) within lipid droplets serving as heat insulator and a buffer against lipotoxicity from free fatty acids [14], [15], [16]. In times of energy restriction, TG hydrolysis (lipolysis) and subsequent fatty acid is utilized to provide energy. However, BAT is the site of energy dissipation linked to heat production (thermogenesis) [17]. In addition, the effects of pika leptin on energy expenditure are also reflected in a reduction in the level of plasma glucose, triglyceride, total cholesterol and free fatty acids (Table 1). The above data indicate that, like leptin from other species, pika leptin mediates energy homeostasis via reducing energy intake and increasing energy expenditure by means of lipid and glucose metabolism. White and brown adipose tissues are thus the important targets of pika leptin adaptive thermogenesis.

The function of BAT is to produce heat in response to cold temperatures by means of nonshivering adaptive thermogenesis, which is essential for the maintenance of body temperature in small mammals [18]. Here, we investigate thermogenic characteristics in BAT induced by pika leptin under both warm and cold conditions. Pika leptin obviously activates adaptive thermogenesis of brown adipocytes, observed as reduced lipid accumulation, TG content, and increased mitochondrial biosynthesis. In general, fatty acid oxidation in mitochondrial matrices is an important pathway for the release of metabolic energy. In response to energy demands, the fatty acids are mobilized from triacylglycerol lipolysis and used by mitochondria in brown adipocytes. Our findings indicate that pika leptin can increase the capacity of lipid oxidation and activity of brown adipocytes.

To gain insights into the mechanism of adaptive thermogenesis in BAT mediated by pika leptin, expression level of several hallmark genes involved in the regulation of thermogenesis were investigated. In addition, to test the hypothesis concerning adaptive functional evolution of pika leptin, we investigated and compared genes responsible for adaptive thermogenesis in both pika leptin and human leptin. We systemically compared expression levels of genes related to BAT-specific thermogenesis between pika leptin and human leptin. It is widely thought that the sympathetic nervous system (SNS) is the major neuroendocrine efferent pathway in the regulation of thermogenesis. Leptin causes the SNS to release norepinephrine (NE) and stimulates the activity of the hypothalamic-pituitary-thyroid axis to release thyroid hormone (TRH) [19]. NE and TRH primarily act on the β3- adrenergic receptors and thyroid hormone receptors present on the surfaces of mature brown adipocytes to activate hormone-sensitive lipase (HSL), which in turn, increases lipolysis of triglyceride droplets. Intracellular free fatty acids liberated from triglycerides then activate UCP1, which initiates heat production and enters the β-oxidation cycle in the mitochondria as the thermogenic substrate. The thermogenic processes of SNS-BAT-UCP1 are mediated by multiple central and peripheral factors [20]. Binding with the long-form leptin receptors (Ob–Rb) distributed in brain and peripheral tissues is crucial for the intracellular leptin signal transduction. Transducer and activator of transcription 3 (STAT3) is one important signal cascade downstream of leptin receptor signaling [21]. LRb-STAT3 signal is central to both the control of energy expenditure by leptin and the neuroendocrine regulation of the SNS and the thyroid axis [22]. Leptin-induced release of norepinephrine from SNS is via the activation of melanocortin receptor 4 (MCR4) in the hypothalamus [23]. In the present study we found marked differences in expression levels of genes participating in the central regulation of thermogenesis in the hypothalamus. There is good evidence that the ability of pika leptin to induce thermogenesis at the level of the central nervous system is more enhanced than that of human leptin, which is indicated by a dramatic elevation of expression levels of key signal molecules such as STAT3, leading to a 7.9 fold increase compared to human leptin. It is widely reported that food intake is regulated by leptin binding to the hypothalamic long form receptor (Ob–Rb) located on arcuate nucleus (ARC) neurons, which are key targets for circulating leptin: these are the orexigenic (stimulates appetite) NPY and AgRP neuropeptides and the anorexigenic POMC and CART neuropeptides (suppresses appetite). Leptin activates POMC and CART neurons and blocks NPY and AgRP neurons. Furthermore, it has recently been shown that central hypothalamic administration of RANKL and RANK expression results in increased body temperature of female mice and rats [24], [25]. RANKL and RANK refer to the receptor-activator of NF-κB ligand and its tumour necrosis factor (TNF)-family receptor. Here, we offer the possibility that adaptive functional evolution of pika leptin may include a CART promoting RANK-mediated NST response in both male and female pika. Initial support for this hypothesis is a thermogenic response combined with significant up-regulation of RANK in the central nervous system of the male mice treated by pika leptin. From skeletal research, we know that CART inhibition of PKA phosphorylation of ATF-4 has the effect of down-regulating RANKL production and thus decreases osteoblastic differentiation and bone resorption [26]. That CART may have this dual role would be important, because while a central increase in RANKL produces an increase in body temperature, its peripheral manufacturing by osteoblasts to regulate osteoclast differentiation and bone resorption would not be a desirable outcome. It may be highly relevant to note that adiponectin, an insulin sensitizing adipokine, inhibits RANKL production when binding to its osteoblast AdipoR1 receptor [27]. Thus, within one metabolic loop, energy homeostasis is maintained without deleterious consequences to the skeleton. Insulin receptor signaling in osteoblasts controls osteocalcin expression [28] which regulates β-cell proliferation and insulin production, which in turn results in adiponectin production by adipocytes, increasing insulin sensitivity and whole-body glucose metabolism (without undesirable bone resorption), and stimulating increased leptin, central RANKL, and sympathetic drive [27]. Therefore, the above findings suggest that the capacity of induced thermogenesis in central system is more marked in pika than human leptin.

In BAT, expression levels of the key genes involved in surface receptors of brown adipocytes, β-oxidation of fatty acids, mitochondria biosynthesis and “uncoupled” heat production is further investigated and compared between pika leptin and human leptin. In ordinary tissues, degradation of fuel molecules is directly coupled to ATP synthesis, however, in BAT, this coupling is disrupted by uncoupling protein 1 (UCP1) which is specifically expressed in the mitochondria inner membrane. This protein directly causes conversion of driving force of ATP synthesis into heat. UCP1 plays an indispensable role in thermogenesis, and this increase in the expression of the UCP1 gene is suitable for meeting the physiological demand [14], [29]. This process of heat production is mediated by multiple factors. Peroxisome proliferators-activated receptors (PPARs), especially isoforms α and γ, have crucial roles in adipocyte differentiation. In BAT, both PPARs contribute to the expression of the UCP1 gene [30], [31]. PPARγ coactivator 1α (PGC-1a) is a PPARγ interacting protein and is restricted to oxidative tissues including BAT and skeletal muscle [32]. It is observed that cold and /or adrenergic stimulation increases the expression of this gene in BAT and then induces elevated expression of UCP1 [33], [34]. The heart-type fatty acid-binding protein (FABP3) is essential for efficient brown adipose tissue fatty acid oxidation and cold tolerance. Fabp3−/− mice exhibit extreme cold sensitivity despite induction of uncoupling and oxidative genes and hydrolysis of brown adipose tissue lipid stores. Fabp3−/− brown adipocytes fail to oxidize exogenously supplied fatty acids, whereas enhanced Fabp3 expression promotes more efficient oxidation [35], [36]. The orphan nuclear receptor estrogen-related receptor α (ERRα), known as one of the target genes of PGC-1α, induces mitochondrial biogenesis and expression of genes involved in oxidative metabolism that are regulated by the peroxisome proliferator-activated receptor γ coactivators (PGC)-1α and PGC-1β in BAT [37], [38]. Type 2 iodothyronine deiodinase (DIO2) is another effector in response to cold stimulation in BAT. DIO2 converts thyroxine (T4) into triiodothyronine (T3), which activates the thyroid receptor and then enhances the transcription of UCP1 [39], [40]. Elovl3 is a very long-chain fatty acid elongase expressed only in liver and BAT that is strongly induced during cold stimulation, and the resulting brown fat recruitment links this protein to the thermogenic process [41], [42]. Each of these genes having crucial roles in thermogenesis in BAT were investigated here. Our findings show that pika leptin activates thermogenesis in BAT via up-regulating the expression level of UCP1, PGC-1α, FABP3, ERRα, DIO2 and Elovl3. Compared with human leptin, elevated level of these genes by pika leptin is more significant under both warm and cold conditions, which implies a more enhanced ability of pika leptin to induce β-oxidation, mitochondrial biogenesis and heat production than human leptin. This discovery is very important and confirms the hypothesis of adaptive functional evolution of pika leptin to extreme cold. This physiological characteristic enables plateau pika to better adapt to the extremely cold conditions faced on the Qinghai-Tibetan Plateau.

To better understand the thermogenic function of pika leptin systematically, we have provided an overview of the characteristics of adaptive pika leptin evolution by comparing responses to warm and cold conditions. Consistent with most studies, cold exposure significantly increases energy intake and output which is reflected by increased appetite, adaptive thermogenesis, glucose uptake, lipid mobilization and weight loss in both leptin-treated and PBS-control mice. When exposed to cold, endotherms exhibit an enhanced energy demand to overcome hypothermia caused by the increased temperature gradient between internal body and external environment. Leptin treatment also induces adaptive thermogenesis to cold stimulation. Interestingly, leptin treatment combined with cold exposure has a synergistic effect, exhibited as an enhanced capacity of adaptive thermogenesis than either cold stimulation or leptin treatment alone. This capacity is represented as increased mitochondrial protein and expression levels of key genes involved in adaptive thermogenesis in BAT and the hypothalamus, such as UCP1, ERRα, PGC-1α, FABP3, β3-AR, DIO2, ELOVL3, STAT3 and RANK.

In sum, we first prepared recombinant leptin protein from a cold-adapted mammal on the Qinghai-Tibetan Plateau, the plateau pika, and we evaluated the adaptive thermogenesis of pika leptin under both warm and cold acclimation. Our data show that pike leptin can regulate energy homeostasis via reduced food intake and increased energy expenditure under both warm and cold conditions. Compared with the leptin protein from species habitually living at more temperate climes, such as human leptin used in this study, pika leptin exhibits a superior capacity to induce adaptive thermogenesis by means of enhanced β-oxidation, mitochondrial biogenesis and heat production than that of human leptin. Moreover, leptin treatment combined with cold stimulation has significantly more synergistic effect on adaptive thermogenesis than either cold exposure or single leptin treatment alone. Our findings confirm the hypothesis that extreme cold stress on the Qinghai-Tibetan Plateau has driven the functional evolution of pika leptin to enhance adaptive thermogenesis in BAT, which contributes to the plateau pikas' ecological adaptation to and survival in the harsh plateau environment.

## Materials and Methods

### Plasmid construction, expression and purification of recombinant pika leptin

The cDNA of mature plateau pika leptin (Accession Number: DQ983189) without signal peptide sequence was cloned via BamH I/Xho I sites into the pMAL-c2x vector (New England Biolabs, NEB), named as pMAL-c2x-pplep. *Escherichia coli* BL21 (DE) cells contained pMAL-c2x-pplep were grown overnight, shaking at 250 rpm at 37°C in LB broth supplemented with 100 µg/ml ampicillin. Then the culture was diluted in 1 L of the same medium (1:100) and grown with shaking at 250 rpm to mid-log phase (A_600 _nm = 0.4–0.5). IPTG (Isopropyl-β- -thiogalactopyranoside) was added to a final concentration of 1 mM to induce production of the fusion protein. The culture was continuously grown at 25°C with shaking at 250 rpm for 20 hours. Cells were harvested by centrifugation then suspended in buffer A (50 mM HEPES, 500 mM NaCl, 30 mM imidazole, PH 7.6) and sonicated on ice, then centrifuged at 12,000 g for 40 min at 4°C. The supernatant filtered through 0.22 µm was used to purify pika leptin protein by immobilized metal affinity chromatography (IMAC) on the xk 16 column packed with Ni Sepharose 6 Fast Flow (GE Healthcare, USA) equilibrated in buffer A. The column was washed with 10 column volumes of buffer A and then tobacco etch virus (TEV) protease was added onto the column to cut His_6_-MPB (maltose-binding protein) fusion protein at 4°C for 16 h. The faction only containing PPLEP was eluted by buffer A, while the His_6_-MBP still bound with the Ni column. The PPLEP protein solution was concentrated and exchanged with storage buffer (phosphate buffered saline, PH7.4, containing 10% glycerol, 1 mM DTT, 1 mM EDTA). Aliquots were stored at −80°C prior to further use. All samples were analyzed by 15% sodium dodecyl sulfate (SDS)-polyacrylamide gel electrophoresis (PAGE), and stained with 0.25% Coomassie brilliant blue R250. The method of Bradford was used to determine the protein content with BSA as the standard. The recombinant human leptin protein was acquired via the same way as pika leptin described above.

### Characteristics of adaptive thermogenesis of pika leptin in brown adipose tissue under both warm and cold acclimation conditions

#### Animals

The use of the animals was in agreement with the Animal Care and Use Committee of the Hebei Medical University (permit number: 1011145). Male C57BL/6J mice weighing 20–25 g were used and housed in individual cages, fed standard laboratory chow and drank water ad libitum. The mice were exposed with artificial dark-light cycles (lights on from 0700 to 1900 h) in either cold (5±1°C) or warm (23±1°C) temperature.

### Experiment design

#### Experiment 1 Effects of intraperitoneal administration of pika leptin on thermogenesis in BAT under warm acclimation

After 7 days of habitation to the room condition (23±1°C), male C57BL/6J mice were randomly divided into two groups (n = 8 per group): WL (pika leptin treatment) and WP (treated with PBS buffer). Mice of WL received twice daily intraperitoneal injection of pika leptin for 7 consecutive days. WP group mice received equal volume PBS injection. The dose of recombinant leptin was 5 µg/g body weight per day. Body weight and food intake were recorded daily. At the end of the experiment, animals were deprived of food for 12 h then decapitated under light ether anesthesia between 0800 and 0900 AM. Blood serum was collected. The heart, liver, spleen, lung, kidneys, brown adipose tissue (BAT) depot (interscapular) and white adipose tissue (WAT) depot (epididymal) were removed, weighed and rapidly taken, frozen in liquid nitrogen and stored at −80°C until analyzed.

#### Experiment 2 Effects of intraperitoneal administration of plateau pika leptin on thermogenesis in BAT under cold acclimation

To explore the effects of pika leptin on adaptive thermogenesis in cold conditions, male C57BL/6J mice were maintained at 5±1°C for 28 days to habituate them to chronic cold exposure, and then divided into two groups (n = 8): CL (pika leptin treatment) and CP (treated with PBS buffer). The dose of injection was 5 µg/g body weight per day. After 7 consecutive days of treatment of pika leptin at 5±1°C, the mice were killed and sampled as described in experiment 1.

#### Experiment 3 Comparison of expression level of hallmark genes related to adaptive thermogenesis in BAT and hypothalamus between pika leptin and human leptin under both warm and cold acclimation

To confirm the hypothesis of adaptive function evolution of pika leptin, differences of expression levels of genes related to adaptive thermogenesis between pika leptin and human leptin were determined. The conditions of animal feeding and treatment were the same as those performed in experiment 1 and experiment 2. At the end of the experiment, the hypothalamus and BAT were rapidly taken and frozen in liquid nitrogen and stored at −80°C for further analysis.

### Quantitative Real-Time RT-PCR assay of mRNA expression of hallmark genes in BAT and hypothalamus

Total RNA was isolated from interscapular BAT or the hypothalamus by using RNAiso Plus Reagent (cat. No. D9108A, Takara Biotechnology (Dalian) Co, Ltd) according to the manufacturer's protocol. RNA samples were treated with RNase-free DNase I to denature the contamination of genomic DNA. 500 ng of total RNA was transcribed into first-strand cDNA for each sample by using reverse transcription kit (cat. No. DRR037A, Takara Biotechnology (Dalian) Co, Ltd) according to the manufacturer's instruction. Gene expression was assessed by quantitative PCR by using gene-specific primers (See Table S1, which is published as supporting information on the PLoS One web site.) and SYBR Green I qPCR kit (cat. no. DRR041D; Takara Bio, Otsu, Shiga, Japan) in the Rotor-Gene ™ 6000 real-time rotary analyzer (Corbett Research Pty Ltd). Each sample was analyzed in triplicate. At the end of the amplication, melting curve analysis showed whether there were nonspecific amplications. mRNA expression data were normalized by using β-actin as a reference gene.

### Morphological analysis of WAT and BAT

For histological analysis, epididymal WAT and interscapular BAT were dissected, washed with PBS, fixed overnight in 4% paraformldehyde, and embedded in paraffin for sectioning. Eight- to 10-µm sections were stained with hematoxylin/eosin.

### Ultrastructure analysis of BAT by transmission electron microscopy

Interscapular BAT was cut into small pieces (≈1 mm^2^), and fixed overnight with 2% paraformaldehyde in 0.1 M phosphate buffer (PH 7.4). Samples were then washed in 0.1 M sodium cacodylate buffer and postfixed for 5 h with 1% OsO4 in cacodylate buffer. Samples were dehydrated in a graded ethanol series and embedded in Epon/Araldite resin overnight. Ultrathin sections were obtained and examined with a Hitachi H-7500 microscope.

### Mitochondrial protein quantification of BAT

Interscapular BAT depots were homogenized by using a Potter-Elvehjem tissue homogenizer in buffer A (0.25 M sucrose/0.1 M Tris-Cl/1 M KCl, PH 7.4) and centrifuged at 3500× g for 10 min at 4°C. The supernatant was further centrifuged at 12000× g for 30 min at 4°C. Then the supernatant was used to quantify total protein content of BAT. The pellet containing mitochondria was washed once and re-suspended in buffer B (20 mM Tris-Cl/2 mM EDTA/100 mM KCl, PH 7.4) and then mitochondrial protein in BAT was quantified. Protein concentration was measured by using the Bradford method.

### Content of triglyceride in BAT

BAT lipids were extracted in a mixture of 2:1 chloroform/methanol and washed once with PBS buffer (phosphate buffered saline, PH7.4). BAT triglycerides were measured by using triglycerides kit (BioSino Bio-technology & Science Inc).

### Plasma Measurements

Plasma glucose was measured using the glucose oxidase glucose assay kit (BioSino Bio-technology & Science Inc). Plasma TG and cholesterol content were measured using Triglycerides Kit and Cholesterol Kit (BioSino Bio-technology & Science Inc). Plasma fatty acids were measured using the fatty acid assay kit (Nanjing Jiancheng Bioengineering Institute).

### Statistical analysis

Results were presented as mean ± SEM and were evaluated by using Student's t test for two groups, ANOVA for three groups and by repeated measure ANOVA for analysis of body weight and food intake.

## Supporting Information

Table S1 Primers used in the study.
(XLS)
